# Narrative Medicine to integrate patients’, caregivers’ and clinicians’ migraine experiences: the DRONE multicentre project

**DOI:** 10.1007/s10072-021-05227-w

**Published:** 2021-04-15

**Authors:** Maria Clara Tonini, Alessandra Fiorencis, Rosario Iannacchero, Mauro Zampolini, Antonietta Cappuccio, Raffaella Raddino, Elisabetta Grillo, Maria Albanese, Gianni Allais, Marco André Bassano, Filippo Brighina, Terenzio Carboni, Fabio Frediani, Licia Grazzi, Carmela Mastrandrea, Franca Moschiano, Maria Gabriella Poeta, Angelo Ranieri, Renato Turrini, Maria Giulia Marini

**Affiliations:** 1 Centre for Diagnosis and Treatment of Headache, Clinica San Carlo, Paderno Dugnano, MI Italy; 2 Healthcare Area, ISTUD Foundation, via Paolo Lomazzo 19, 20124 Milan, Italy; 3 Department Neurology Headache Center, Hospital Regional “Pugliese-Ciaccio”, Catanzaro, Italy; 4 USL Umbria 2, Department of Rehabilitation, San Giovanni Battista Hospital, Foligno, PG Italy; 5 Medical Department, Novartis Farma, Origgio, Italy; 6 Regional Referral Headache Center, Neurology Unit, Department of Systems Medicine, University Hospital of Rome “Tor Vergata”, Rome, Italy; 7 Women’s Headache Center, Department of Surgical Sciences, University of Turin, Turin, Italy; 8 Department of Biomedicine, Neuroscience and Advanced Diagnostic (BIND), University of Palermo, Palermo, Italy; 9 Division of Neurology, Madonna del Soccorso Hospital, San Benedetto del Tronto, AP Italy; 10 Headache Centre, Neurological Department, San Carlo Borromeo Hospital, ASST Santi Paolo e Carlo, Milan, Italy; 11 Headache Centre, Carlo Besta Neurological Institute and Foundation, Milan, Italy; 12 Division of Neurology and Stroke Unit, CTO Hospital, Naples, Italy; 13 Headache Centre, Neurology Unit, Galliera Hospital Genova, Genoa, Italy; 14 Division of Neurology and Stroke Unit, A. Cardarelli Hospital, Naples, Italy

**Keywords:** Migraine, Narrative medicine, Doctor-patient relationship, Illness experience, Quality of life

## Abstract

**Background:**

Although migraine is widespread and disabling, stigmatisation and poor awareness of the condition still represent barriers to effective care; furthermore, research on migraine individual and social impact must be enhanced to unveil neglected issues, such as caregiving burden. The project investigated the migraine illness experience through Narrative Medicine (NM) to understand daily life, needs and personal resources of migraneurs, their caregivers and clinicians, and to provide insights for clinical practice.

**Methods:**

The project involved 13 Italian headache centres and targeted migraneurs, their caregivers and migraine specialists at these centres. Written narratives, composed by a sociodemographic survey and illness plot or parallel chart, were collected through the project’s webpage. Illness plots and parallel charts employed open words to encourage participants’ expression. Narratives were analysed through Nvivo software, interpretive coding and NM classifications.

**Results:**

One hundred and seven narratives were collected from patients and 26 from caregivers, as well as 45 parallel charts from clinicians. The analysis revealed migraine perception in social, domestic and work life within the care pathway evolution and a bond between *chaos* narratives and day loss due to migraine; furthermore, narratives suggested the extent of the caregiving burden and a risk of underestimation of migraine burden in patients’ and caregivers’ life.

**Conclusion:**

The project represents the first investigation on migraine illness experience through NM simultaneously considering migraneurs’, caregivers’ and clinicians’ perspectives. Comparing narratives and parallel charts allowed to obtain suggestions for clinical practice, while NM emerged as able to foster the pursuing of migraine knowledge and awareness.

**Supplementary Information:**

The online version contains supplementary material available at 10.1007/s10072-021-05227-w.

## Introduction

Migraine is a common neurological disorder, defined as a recurrent primary headache disorder [1]; it is experienced by the 14.7% of the global population [2] and has a lifetime incidence three times higher among women than men [3]. Migraine can vary in intensity and severity and is characterised by intolerable pain aggravated by movement and frequently accompanied by nausea, vomiting, photophobia, phonophobia and significant disturbance of usual activities [1]; attacks evolve within 4–72 h and commonly involve a premonitory, headache, and postdrome phase, while one-third of migraineurs experience an aura phase with reversible neurological symptoms [4, 5].

The primary prophylactic treatments in migraine are not specific as they have been designed for other diseases and then addressed to migraine [6], with a low treatment adherence and tolerability issues [7]; thus, new therapies addressed to modulate the Calcitonin Gene Related Peptide (CGRP) activity have opened a promising scenario for both acute and preventive treatment [8–10].

Although migraine is widespread and disabling, stigmatisation and poor awareness of the condition represent two barriers to effective treatment. Moreover, migraine is still misdiagnosed by non-specialised professionals and undertreated: a significant part of migraine patients has never consulted a healthcare specialist or has never been diagnosed, while undergoing unnecessary medical imaging, receiving inappropriate treatments, or self-treating [11–16].

Against this scenario, a broader culture of prevention and a multidisciplinary approach to migraine must integrate the clinical pathway [17]. Furthermore, research on migraine social impact must be enhanced to unveil neglected issues, such as caregiving experience [18]: even if studies in migraineur quality of life have increased [19, 20], the multifaceted burden of migraine [18] has leaded to fragmented results and interventions.

Narrative research has been addressed by the World Health Organisation (WHO) as informative for quality-of-life investigations in leading clinical practice [21]. In particular, Narrative Medicine (NM), based on illness narratives [22], pursues to integrate the *disease*-centred and biomedical approach with the *illness*-centred and *sickness*-centred approaches, respectively looking at the individual experience and the social meaning of a condition [23]. In research, NM indicates potential interventions on a specific condition through integrating the perspectives of all actors involved in the care pathway [24]. Results deriving from NM have been increasingly employed by scientific societies and healthcare facilities to improve the efficacy of healthcare services and quality of care [25].

The NM project “DRONE – Inside the Research: Observatory on Migraine Narratives” aimed to investigate the migraine illness experience by employing the analysis of narratives (a) to understand daily life, real needs and personal resources of migraineurs, their caregivers and clinicians within the evolution of the care pathway, and by doing so (b) providing insights to foster clinical practice as well as migraine knowledge and care. Although other narrative researches concerned the migraine illness experience [26, 27], this is the first project to address personal, relational and care aspects of migraine by considering at the same time these three different points of view.

## Methods

### Research design and setting

The project was conducted in Italy between December 2019 and October 2020 and targeted migraine patients, their caregivers, and expert clinicians in headache disorders. Thirteen headache centres were selected (Supplement 1), equally distributed among Northern, Central, and Southern Italy. In February 2020, clinicians from these centres underwent a webinar conducted by scholars from ISTUD Foundation to be trained in NM and on the project’s purposes, design and data collection tools. The clinicians were then invited to engage patients and their caregivers to join the research through the dedicated webpage www.medicinanarrativa.eu/drone.

A migraine diagnosis or the caregiving of a migraineur represented the eligibility criteria for patients and caregivers, as well the willingness to share by writing their illness experience; however, the ability to communicate in Italian was indispensable for the inclusion in the project.

### Data collection

Written narratives were collected anonymously through the project’s webpage; patients described in parallel charts could not coincide with those who shared their experience. Next, raw and anonymous narratives were downloaded as a Microsoft Excel spreadsheet. A sociodemographic survey constituted the written narrative for patients and caregivers, together with an illness plot [28] aimed to chronologically guide the narrative to identify evolutions over time and characterised by evocative and open words to encourage individual expression [29]. Furthermore, parallel chart [22, 30] was addressed to collect clinicians’ experience: it constitutes a personal notebook to write reflections and feelings in plain language in addition to the technical reports of clinical chart [31]. These narrative tools (Supplement 2) were specifically designed for the three groups of participants while addressing common aspects: (a) the personal and social experience of migraine, (b) migraine management and the care pathway, and (c) the daily living with migraine, with a particular focus on work, domestic and relational spheres.

Data collection tools were created by three ISTUD researchers, different for academic backgrounds, and then reviewed within the project Steering Committee, involving three professionals in headache disorders, to reduce the cognitive bias.

### Ethical considerations

The project was performed according to the Declaration of Helsinki. Before their involvement, participants provided web-based informed consent after being briefed on the project’s purposes and confidential data handling procedures, according to the Italian Law 196/2003 [32] and the General Data Protection Regulation of the European Union 2016/679 [33]. The Ethical Committee of the University Hospital of Rome Tor Vergata (Rome, Italy) approved the project in April 2020.

### Analysis

ISTUD researchers analysed the sociodemographic survey through descriptive statistics; no question was mandatory. Anonymous narratives were entered into NVivo software for coding and analysis [34]. Ten narratives were collectively coded for each group to assess consistency across team members; afterwards, each narrative was coded separately and then reviewed within weekly peer debriefings to limit bias in the interpretation.

Researchers employed open interpretive coding to identify and analyse emerging topics, and retrospectively applied two classifications to the analysis of narratives:

- Kleinman’s classification [23], which distinguishes among *disease*-, *illness*-, and *sickness*-related aspects in narratives, respectively concerning the biomedical description of the condition, its personal experience and its social perception.
- Frank’s classification [35], which identifies *chaos* narratives, i.e. anti-narratives revealing vulnerability of the narrator, *restitution* narratives, i.e. when the narrator explores the care pathway experience and meaning, and *quest* narratives, i.e. when the condition is lived as a motivation for change.

Moreover, we asked participants to describe migraine through a metaphor, attempting to trace spontaneous meaning associations created through daily language.

Results from the analysis were shared with the Steering Committee to address emerged topics and data interpretation collectively.

## Results

### Sociodemographic aspects

One hundred and seven migraineurs and 26 caregivers (patients’ partners in 56% of cases) participated in the project, as well as 14 clinicians who wrote 45 parallel charts. Table 1 summarises participants’ sociodemographic data and includes non-responses as a separate category.

**Table 1:** Sociodemographic data of participants

	Patients (*N* = 107)	Caregivers (*N* = 26)	Patients in parallel charts (*N* = 45)	Clinicians (*N* = 14)
Gender				
Women	89 (83%)	11 (42%)	37 (82%)	6 (43%)
Men	18 (17%)	15 (58%)	8 (18%)	8 (57%)
Average age (yrs)				
Mean (min-max)	47 (16-80)	47 (26-77)	45 (12-80)	57 (37-68)
Nationality				
Italian	105 (98%)	26 (100%)	45 (100%)	14 (100%)
European	1 (1%)	–	–	–
Extra-European	1 (1%)	–	–	–
Geographic residence				
Northern Italy	49 (46%)	15 (58%)	22 (49%)	4 (29%)
Central Italy	24 (22%)	6 (23%)	11 (24%)	6 (42%)
Southern Italy	33 (31%)	4 (15%)	12 (27%)	4 (29%)
Nonresponses	1 (1%)	1 (4%)	–	–
Education				
Elementary school	-	-	1 (2%)	–
Middle school	9 (8%)	2 (8%)	6 (14%)	–
High school	45 (42%)	12 (46%)	19 (42%)	–
Bachelor/Master	52 (49%)	11 (42%)	19 (42%)	–
Nonresponses	1 (1%)	1 (4%)	-	–
Employment status				
Student	9 (8%)	–	4 (9%)	–
Working	79 (75%)	15 (58%)	32 (71%)	–
Not working	9 (8%)	2 (8%)	5 (11%)	–
Retired	8 (7%)	6 (23%)	4 (9%)	–
Nonresponses	2 (2%)	3 (11%)	–	–
Marital state				
Single	65 (61%)	2 (8%)	16 (36%)	–
Married	34 (31%)	20 (77%)	24 (53%)	–
Separated	7 (7%)	3 (11%)	4 (9%)	–
Nonresponses	1 (1%)	1 (4%)	1 (2%)	–
Professional activity (yrs)				
Mean (min-max)	–	–	–	30 (11-40)
Specialisation				
Pharmacology	–	–	–	1 (7%)
Neuropathology	–	–	–	1 (7%)
Neurology	–	–	–	12 (86%)
Workplace				
Hospital	–	–	–	5 (36%)
University Hospital	–	–	–	3 (21%)
Local Health Authority	–	–	–	5 (36%)
Private practice	–	–	–	1 (7%)

Data presented as N (%) or mean (minimum-maximum)

**Fig. 1:**
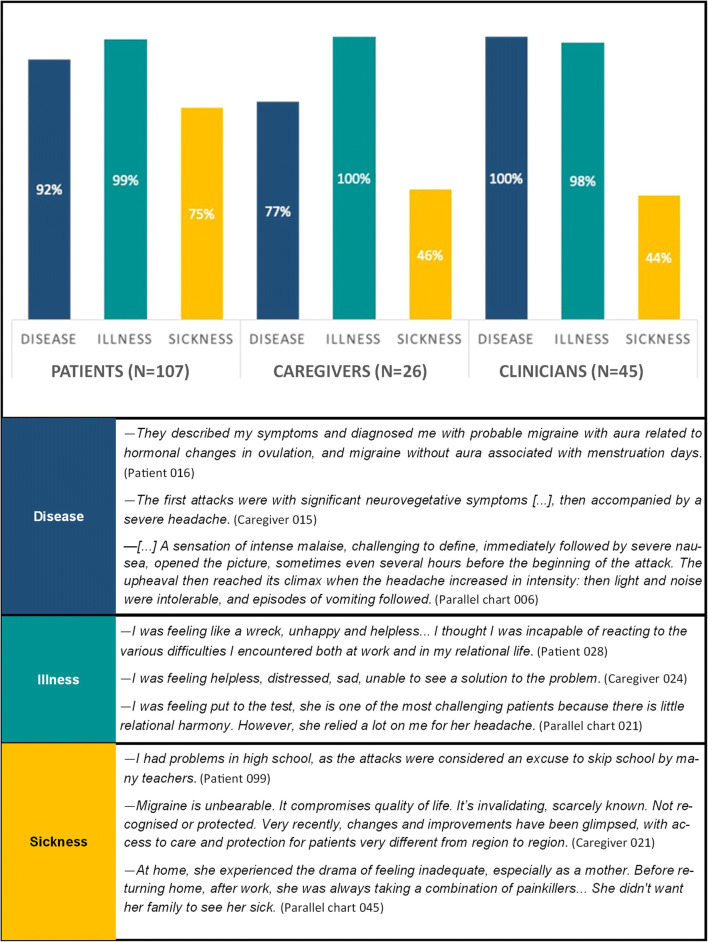
Disease-, illness, and sickness-related aspects: distribution and quotes from narratives

**Fig. 2:**
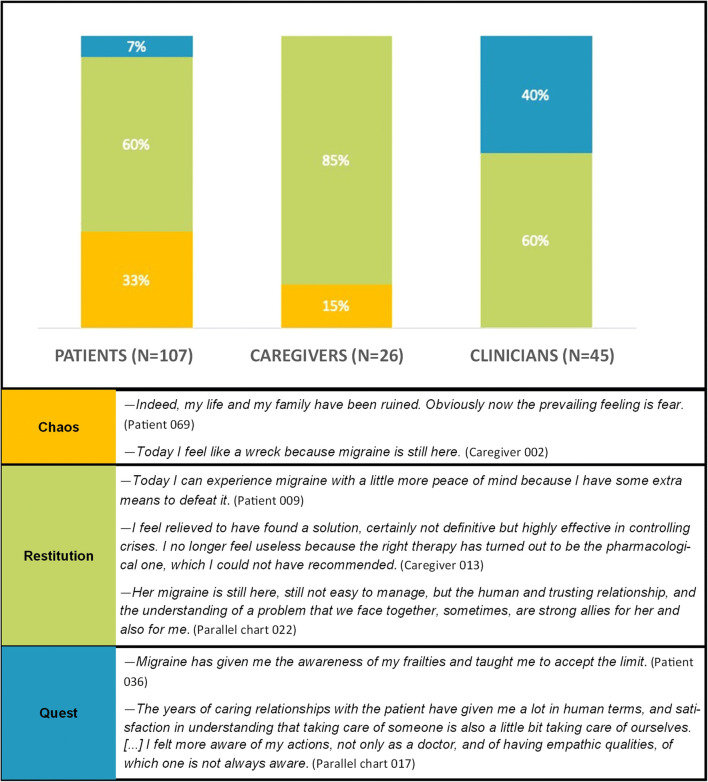
Chaos, restitution and quest narratives: distribution and quotes from narratives

**Fig. 3:**
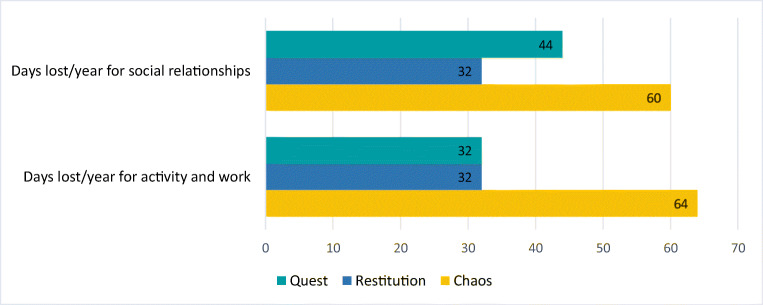
Average lost days per year for activity/work and social relationships compared to Frank’s narrative classification

**Fig. 4:**
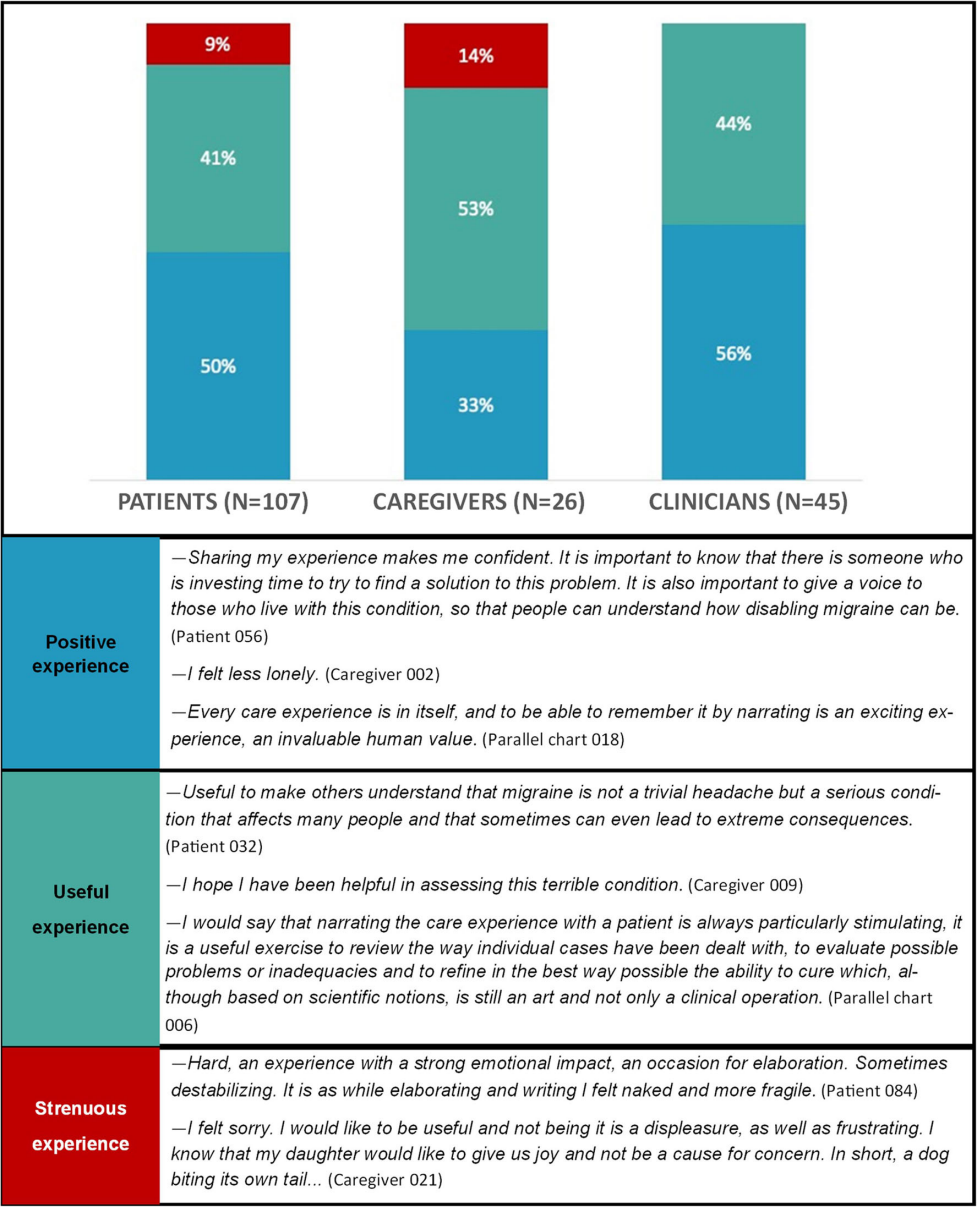
The narrating experience for participants: distribution and quotes from narratives

Results are presented by following three main lines: (a) migraine illness experience analysed through NM classifications and metaphors; (b) migraine management and the evolution of care relationship; (c) migraine impact on activity, work and relational spheres. Figures 1, 2, 3, and 4 and Tables 2 and 4 provide quotes from narratives, while three extracts from participants’ narratives are available in Supplement 3; applied codes differ from those used to identify participants to reduce the risk of re-identification.

**Table 2:** Living with migraine

	Patients (*N* = 107)	Caregivers (*N* = 26)	Patients in parallel charts (*N* = 45)
Average migraine duration (yrs)			
Mean (min-max)	27 (2-60)	27 (0-50)	21 (1-65)
Average episodes in a month			
Mean (min-max)	12 (0-31)	12 (0-31)	6 (1-25)
Specialist who made the diagnosis			
Migraine specialist	79 (74%)	–	–
Neurologist	19 (18%)	–	–
General practitioner	3 (3%)	–	–
Pharmacist	2 (2%)	–	–
Not answered	4 (4%)	–	–
Family member suffering of migraine			
Yes	78 (73%)	–	–
No	27 (25%)	–	–
Not answered	2 (2%)	–	–

Data presented as N (%) or mean (minimum-maximum)

### The migraine experience through narratives and metaphors

Globally, almost all participants focused on *illness*-related facets [23] in narrating their stories, while technical and clinical language is less present in caregiver narratives (Fig. 1). *Sickness*-related aspects similarly emerged in 46% of caregiver narratives and 44% of parallel charts but were more remarkably present within patient narratives (75%), where incomprehension, inadequacy and sense of being discriminated represented three spontaneously emerged issues.

*Restitution* narratives [35] were prevalent among the three groups (Fig. 2). Caregivers reported no *quest* narratives, compared to 40% of clinicians and 7% of patients. *Chaos* narratives were lacking in parallel charts, while characterised 33% of patient and 15% of caregiver experiences.

Metaphors were clustered into four main thematic groups: (a) metaphors related to limitation and still life, mostly employed by caregivers (38%); (b) metaphors concerning malignant nature or entity, mainly used by patients (31%) and clinicians (30%); (c) metaphors referring the action of a hammer, pressure or pulsing, reported in particular by patients (30%) and clinicians (34%); (d) metaphors denoting stabbing and pain mostly employed by caregivers (25%); Supplement 4 provides distribution and examples of these four groups of metaphors. Only the remaining 5% of parallel charts reported positive or neutral metaphors to describe migraine (—*My patient’s migraine could be described as an old friend*, parallel chart 041).

### Migraine management and care relationship

Within the survey, patients reported to have suffered from migraine, on average, from 27 years and to have 12 migraine episodes on average in a month; 74% received the diagnosis at the reference headache centre and 73% declared to have one or more family members suffering from migraine (Table 2).

As for migraine management previous to the current care pathway, three main issues spontaneously emerged from narratives: (a) 49% of patients’ narratives referred to a lack of confidence in previous clinicians, also reported in 31% of caregiver narratives and parallel charts; (b) 48% of patients reported the attitude to self-treat, as emerged in 8% of caregiver narratives and 40% of parallel charts; (c) 35% of patients declared to have overused medications for migraine management.

On the opposite, once the patients reached the headache centres, narratives showed a positive therapeutic pathway: 60% of patients and 58% of caregivers defined current therapies as effective, and respectively 25% and 21% stated they represent hope for a better quality of life; still, 15% of patients and 21% of caregivers reported that current therapies have no efficacy. Conversely, only 9% of parallel charts reported treatment inefficacy.

Indeed, care relationships showed to evolve positively: patients’ mistrust in healthcare professionals decreased from 56 to 5%, conversely trust increased from 44 to 95%. In parallel charts, clinicians indicated which communication and care strategies leaded them through the care pathway (Table 3). Beyond proposing (38%) and sharing a therapeutic path (43%), inviting patients to talk about their emotional state (24%) and listening to their migraine stories (36%) emerged as the leading strategies respectively in communication and care. Furthermore, in parallel charts, going beyond the clinical datum (37%) and actively listening and collaborating with patients emerged as the two main learnt attitudes during the care relationship. At the same time, the sense of improving patient quality of life (61%) and gratification (29%) represented the two main feelings reported by clinicians; in comparison, 33% of parallel charts indicated discomfort and impotence at the beginning of the care relationship. Furthermore, when asked within the survey, clinicians reported that their patients consider them as a reference point (65%), relief source (21%) and guardian angel (14%).

**Table 3:** Clinicians’ strategies and learnt attitudes: distribution and quotes from narratives

Communication strategies	Proposing therapeutic options (38%)	—*I told her that we would have tried a new treatment and that I would have committed myself to improve her quality of life*. (Parallel chart 021)
	Inviting patients to share their feelings (24%)	—*I tried to understand her discomfort, sharing her emotional state*. (Parallel chart 003)
	Inviting to a therapeutic alliance (19%)	—*I told her that we would go a long way together, that she would grow up and that I would help her to live in the most "normal" way possible, despite migraine*. (Parallel chart 020)
	Fostering awareness (19%)	—*I tried to reassure him; the condition was not invincible. It was enough to have some tricks: starting the therapy as soon as possible, at the first symptoms; avoiding, as far as possible, known triggering factors such as loss of sleep and alcohol*. (Parallel chart 006)
Care strategies	Sharing the therapeutic path (43%)	—*I made her an appointment for the next day and put her last on the list, I knew the visit would have lasted an eternity! We analysed together the course of her migraine over time, the triggering and predisposing factors, the therapies used with risks and benefits obtained, her lifestyle, her "fears", her "false convictions", her mistakes... In the end, I clearly showed her what for me could be an "innovative" treatment programme, involving not only drugs but also psychological support*. (Parallel chart 045)
	Listening to patient migraine stories (36%)	—*I let her talk about her anger towards doctors and the condition, her physical and psychological pain, her difficulties with family and at work. I explained what migraine is and its implications in everyday life. [...] I gave her a diary to record the attacks, the symptoms and to note down the triggering factors. [...] I let her come to the clinic after hours when she was feeling down or when she had doubts about the new therapy*. (Parallel chart 001)
	Fostering correct information (21%)	—*I gave her all the indications to try to control her headaches, averting severe crises, using ad hoc drugs that nip the pain in the bud, ensuring participation in sports and relational life. I also tried to involve and support the family, especially the mother, in the management of the little patient*. (Parallel chart 042)
Learnt attitudes	Going beyond clinical issues (37%)	—*From this patient, but also from every patient, I learnt that we must go beyond the clinical history. We must never stop at the few words or the few elements that a patient provides to make a diagnosis. We must understand the complexity of a symptom that hides emotional problems, discomfort and even loneliness. We must not be in a hurry. The age of a person, young or old, does not count in the care relationship*. (Parallel chart 018)
	Listening and collaborating with patients (35%)	—*[...] The most essential weapon, which cannot be touched or measured, in the daily fight against disease, is the doctor-patient alliance: you are not alone, everyone does their part, as a team*. (Parallel chart 040)
	Having more patience (16%)	—*I have learned to go beyond appearances, to be patient in moments of discouragement, to give myself and to her another chance; I have learned to cultivate hope*. (Parallel chart 002)
	Aptness of care (12%)	—*Data collection, interaction with the patient and evidence of limitations that the patient experiences are very useful in identifying treatment*. (Parallel chart 019)

### Living with migraine in relational and work context

In the survey, 90% of patients stated that migraine negatively affected their domestic and work activities. Patients were asked to provide, on average, how many days of social and work activities they annually lose because of migraine. Considering these data together with narratives classified according to Frank [35], patients with *chaos* narratives revealed to have lost, annually, 64 days for domestic and work activities and 60 days for social activities, significantly differing from patients with *restitution* or *quest* narratives (Fig. 3).

In narratives, patients reported an overall improvement in social, domestic and work spheres (Table 4) along with the evolution of the care relationship; nonetheless, patients and of caregivers still experienced difficulties in relationships (respectively 27% and 23%) and fatigue in activities (35%). Notably, clinicians in parallel chart described patients that were able to recover in 92% of cases highlighting a strong difference in what is perceived as improvement by specialists and by patients and their caregivers.

**Table 4:** Improvements in activity, work and social spheres: distribution and quotes from narratives

Improvements in domestic and work activities	65% of patient narratives	—*I continued to work, to care for my children. To do so, I was always making medicines varied. I started with nausea, discomfort, vomiting, tiredness, and I felt dull, without wanting to do anything. Then, when migraine became uncontrollable, I just wanted to get into the bed, holding my head and waiting for the effect of the intramuscular medication. Today I am active, more concentrated, I perform better, and I can prolong my activity without paying the consequences as before, that is to say, even if I get tired, I don't get migraines.* (Patient 075).
	65% of caregiver narratives	—*For the most part, she gave up any activity other than lying in bed. Now that she has understood when and how to deal with attacks, she can plan her different activities*. (Caregiver 013)
	93% of parallel charts	*- The patient's work activities are also going better because her concentration has increased and she feels more ready in solving even difficult tasks, she is eating well, exercising and using fewer symptomatic medications.* (Parallel Chart 010)
Relational and social activities	73% of patient narratives	—*Before the attack, I was irritable, so the relationships at that time are compromised. In the days when the attacks are present, I cannot relate because the symptomatology forces me to isolate myself and stay in bed. [...] Today, I can also relate with others because I no longer see myself forced to be isolated and in the dark.* (Patient 078)
	77% of caregiver narratives	—*I felt and worried about our future life. She felt sick. [...] Today I feel decidedly happy, we have found our everyday life again. We go out for dinners and trips as we did twenty years ago, with an even more willing spirit. She feels very well, both physically and mentally.* (Caregiver 007)

Furthermore, in narratives, 67% of patients and 86% of caregivers reported that migraine negatively impacted their quality of life in terms of time and energy loss; nevertheless, migraine emerged as a stimulus to improve self-awareness for 51% of patients and 41% of caregivers.

Globally, the experience of writing and sharing their narratives was positive for participants (Fig. 4). In particular, 41% of patients, 53% of caregivers and 44% of clinicians referred to a sense of being useful for other people dealing with migraine; still, for 9% of patients and 14% of caregivers, the experience was difficult and challenging.

## Discussion

The DRONE project represented the first effort to investigate the migraine illness experience in Italy through NM by considering, at the same time, the perspectives of migraineurs, their caregivers and clinicians.

In patients’ narratives, the coexistence of *disease* and *illness* dimensions [23] highlighted the bound between the clinical evolution of migraine and its individual and emotional experience: in this sense, narratives invite us to consider this condition as a psycho-biological unit and to address a global and patient-centred care. At the same time, *sickness*-related aspects in narratives suggest a demand for social and policy intervention: physical discomfort and pain interfere with patients’ relational and work activities, also confirming previous clinical studies reporting a decrease in work performances for migraineurs [36] with a high impact in terms of stigma [37]. NM classifications allowed to combine stigma with spontaneously emerged issues, namely feeling misunderstood, inadequate or not recognised as subject to a disabling condition. In line with what reported in literature [3], most patients are women of working age, at risk to undergo a double burden [18]. Migraineurs experience loneliness in familiar and work contexts, exclusion from the social sphere, and may feel they can rely only on their own migraine management strategies — as also suggested by the tendency, spontaneously emerged, to self-treat. Moreover, having one or more family members suffering from the same condition may influence them to consider migraine as a “normal” condition.

As emerged within the analysis, patients with *chaos* narratives [35] lose more days of social, domestic and work activities compared to those with *restitution* or *quest* narratives. This finding suggests a bond between the day loss and the capability to find a sequence and meaning in migraine experience — in which *chaos* can be intensified by both work absenteeism and presenteeism — and may foster other studies on the work impact of the condition [38].

Caregiver narratives are predominantly *illness*-centred and highlight the impotence and discomfort, as also suggested by the presence of *chaos* narratives. Caregivers, mostly patients’ partners, and whose participation is lower than in other NM projects [39], show to be deeply dedicated to their loved ones but do not mention their strategies and resources to deal with migraine experience; thus, *sickness*-related issues show that they also suffer from migraine social fallout, in line with studies urging to report migraine impact on caregiving [18]. Furthermore, caregivers’ spontaneous meaning association through metaphors reveals their involvement in understanding patients’ experience, as well for clinicians.

Findings from parallel charts show that an empathic relationship and therapeutic alliance can improve the care pathway and patient quality of life. Migraine specialists emerge as attentive to listening and communication, highlighting how migraine treatment should be considered not only a therapeutic outcome, but also (a) as integrated in a broader *migraine culture*, involving care humanisation and aptness [40], the centrality of therapeutic alliance and patient awareness, and (b) inclusive towards migraine illness and social experience of patients.

Data and narratives report positive evolution of care pathways. Nonetheless, a few issues need to be addressed as potential suggestions for migraine specialists:

- There is a mistrust towards healthcare professionals and an abuse of self-medication in patients who suffer of migraine. Patients found relief when started their care pathway with the specialists of the headache centres, but they are still few compared to the amount of people affected by this disease. The underestimation and the sickness related to migraine are a strong barrier to the “on time treatment”.
- Findings suggest a misalignment towards what clinicians, on the one side, and patients and caregivers, on the other side, consider as a positive evolution of the therapeutic pathway, as well as an improvement in relational and work spheres; if a misalignment can be reported by patients involved in a care pathway, it may arise to an even greater extent in patients who are discontinuously or not followed up. Consequently, the invitation may be that of evaluating the risk of underestimation of the criticalities concerning the migraine experience. Addressing patients’ emotional and social issues may help professionals in this path.
- Since *chaos* narratives result as linked to challenging situations in terms of day loss, professionals may support patients towards *restitution* and *quest* narratives, also inviting them to consider migraine more as an ally than an enemy, as a stimulus towards self-knowledge and awareness, better lifestyle and prevention.
- Caregiving burden is often neglected; nonetheless, caregivers participate to the patients’ migraine experience: they also need empathy and to be supported in finding personal resources to overcome fatigue.

These suggestions also indicate a limitation of the project: since narratives were anonymous, we are not able to precisely state the misalignment perceived between patients and caregivers on one side, and clinicians on the other side. Moreover, we involved only patients already attending headache centres and their caregivers: further investigations are needed (a) to intercept migraineurs who have not yet accessed these centres, as well as a broader number of caregivers, and (b) to examine to a greater extent issues spontaneously emerged. Finally, data collection phase partially corresponded to the lockdown measures decided by the Italian government to contain the Sars-Cov-2 pandemic spread which had consequence not only on the management of the disease and the participation to the project but on patients themselves [41].

## Conclusion

The DRONE project aimed to investigate migraine illness experience to understand daily life, needs and personal resources of migraineurs, their caregivers and clinicians within the evolution of the care pathway, and represented the first Italian project to simultaneously addressing these three perspectives, integrating them and giving voice to this condition in terms of identity and dignity.

Narrative emerged as crucial for in-depth analysis and self-knowledge, while reconnecting the migraine physical experience to emotional and social issues concerning this condition. Comparing narratives and parallel charts allowed to obtain suggestions for clinical practice and insights for migraine knowledge.

NM allowed to foster the pursuing of a migraine *culture and awareness* encompassing patients, caregivers, neurologists, and other healthcare providers up to general practitioners, to acknowledge the burden and address the stigma peculiar to this condition.

## Supplementary Information

ESM 1 (PDF 81 kb)

ESM 2 (PDF 111 kb)

ESM 3 (PDF 99 kb)

ESM 4 (PDF 126 kb)

## Data Availability

All datasets used and analysed during the current research are available in Italian from the corresponding author upon reasonable request.

## Abbreviations

WHO: World Health Organisation
NM: Narrative Medicine
