# Supplementary material for: Narrative Medicine to integrate patients’, caregivers’ and clinicians’ migraine experiences: the DRONE multicentre project
Source: Neurol Sci. 2021 Apr 15;42(12):5277–88. doi: 10.1007/s10072-021-05227-w (PMC8047556; doi:10.1007/s10072-021-05227-w)
Supplement: Supplementary file 1 — (PDF 81 kb) [file 10072_2021_5227_MOESM1_ESM.pdf]

# Narrative Medicine to integrate patients', caregivers' and clinicians' migraine experiences: the DRONE multicentre project.

**Journal:** *Neurological Sciences*

Maria Clara Tonini, Alessandra Fiorencis*, Rosario Iannacchero, Mauro Zampolini, Antonietta Cappuccio, Raffaella Raddino, Elisabetta Grillo, Maria Albanese, Gianni Allais, Marco André Bassano, Filippo Brighina, Terenzio Carboni, Fabio Frediani, Licia Grazzi, Carmela Mastrandrea, Franca Moschiano, Maria Gabriella Poeta, Angelo Ranieri, Renato Turrini, Maria Giulia Marini.

*Corresponding author: Alessandra Fiorencis, Fondazione ISTUD – via Paolo Lomazzo 19, 20124 Milano, Italy. Tel. +39 0323 933 801, Mobile +39 3420499824, e-mail: afiorencis@istud.it. ORCID ID https://orcid.org/0000-0001-9859-5070

## Supplement 1

**Headache centres involved in the DRONE project**

1. Centre for Diagnosis and Treatment of Headache, Clinica San Carlo – Paderno Dugnano (MI), Italy
2. Headache Centre, Carlo Besta Neurological Institute and Foundation – Milan, Italy
3. Headache Centre, Neurological Department, San Carlo Borromeo Hospital, ASST Santi Paolo e Carlo – Milan, Italy
4. Women's Headache Center, Department of Surgical Sciences, University of Turin – Turin, Italy
5. Headache Centre, Neurology Unit, Galliera Hospital Genova – Genoa, Italy
6. Division of Neurology, Madonna del Soccorso Hospital – San Benedetto del Tronto (AP), Italy
7. USL Umbria 2, Department of Rehabilitation, San Giovanni Battista Hospital – Foligno (PG), Italy
8. Regional Referral Centre, Neurology Unit, University Hospital of Rome "Tor Vergata" – Rome, Italy
9. Division of Neurology and Stroke Unit, A. Cardarelli Hospital – Naples, Italy
10. Division of Neurology Unit and Stroke Unit, CTO Hospital – Naples, Italy
11. Neurology Department, Locorotondo Clinic – Locorotondo (BA), Italy
12. Headache Centre, Department of Neurology, "Pugliese-Ciaccio" Regional Hospital – Catanzaro, Italy

13. Department of Biomedicine, Neuroscience and Advanced Diagnostic (BIND), University of Palermo – Palermo, Italy
