# Supplementary material for: Narrative Medicine to integrate patients’, caregivers’ and clinicians’ migraine experiences: the DRONE multicentre project
Source: Neurol Sci. 2021 Apr 15;42(12):5277–88. doi: 10.1007/s10072-021-05227-w (PMC8047556; doi:10.1007/s10072-021-05227-w)
Supplement: Supplementary file 2 — (PDF 111 kb) [file 10072_2021_5227_MOESM2_ESM.pdf]

Narrative Medicine to integrate patients', caregivers' and clinicians' migraine experiences: the DRONE multicentre project.

Journal: *Neurological Sciences*

Maria Clara Tonini, Alessandra Fiorencis*, Rosario Iannacchero, Mauro Zampolini, Antonietta Cappuccio, Raffaella Raddino, Elisabetta Grillo, Maria Albanese, Gianni Allais, Marco André Bassano, Filippo Brighina, Terenzio Carboni, Fabio Frediani, Licia Grazzi, Carmela Mastrandrea, Franca Moschiano, Maria Gabriella Poeta, Angelo Ranieri, Renato Turrini, Maria Giulia Marini.

*Corresponding author: Alessandra Fiorencis, Fondazione ISTUD – via Paolo Lomazzo 19, 20124 Milano, Italy. Tel. +39 0323 933 801, Mobile +39 3420499824, e-mail: afiorencis@istud.it. ORCID ID https://orcid.org/0000-0001-9859-5070

Supplement 2

2.1. Illness plot addressed to people suffering from migraine

Before narrating your experience, could you describe your migraine through an image?
We invite you to narrate your experience with migraine. You can write instinctively and freely, regardless of the form and length of your narrative. Any episode you consider significant will be welcome.

Me, before migraine...
Then something changed, it happened that...
I felt...
The first attacks were...
My body...
During the attacks...
With my family...
With other people...
In my activities...
I wanted...
Living was...
I decided to go for a visit...
The healthcare professionals were...
They told me...
The therapies were...
Today I feel...

Migraine is...
Before a migraine attack...
During the attack...
Today I can...
After...
My body...
With my family...
The others...
In my activities...
I want to...
Living is...
Therapies and healthcare professionals are...
For the future, I would like...
I don't want...
Migraine has taken away...
Migraine gave me...

Thank you for your time, energy and attention. We ask you one last question: How did you feel about writing your experience?

2.2. Illness plot addressed to caregivers

Before you start telling your experience, you could describe your loved one's migraine with an image?
We invite you to narrate your experience with migraine. You can write instinctively and freely, regardless of the form and length of your narrative. Any episode you consider significant will be welcome.

Before the migraine of my loved one...
Then something changed, it happened that...
I felt...
He/she felt...
The first attacks were...
He/she was telling me/not telling me...
During the attacks...
His/her body...
With other people...
In the activities...
I wanted to...
Living was...
He/she decided to go for a visit...
The healthcare professionals were...
They said...
The therapies were...
Today I feel...
Today he/she feels...
Migraine is...
Before a migraine attack...
He/she says...
During the attack...
After the attack...
My body...
The other people...
In the activities...
I want to...
Living is...
Therapies and healthcare professionals are...
For tomorrow I would like...
I don't want...
Migraine has taken us away...
Migraine has given us...

Thank you for your time, energy and attention. We ask you one last question: How did you feel about writing your experience?

2.3. Parallel chart addressed to healthcare professionals

Could you describe your patient's migraine with an image?

We invite you to narrate your experience of treating a person with migraine. You can write instinctively and freely, regardless of the form and length of your narrative. Any episode you consider significant will be welcome.

The first time I saw this person with migraine, I thought...

The patient told me...

During his/her migraine attacks, he told me that he/she was/was not...

His/her body during migraine attacks...

In his/her activities at home...

In his/her activities at work/study...

I felt...

I said...

And I did...

Today this person...

At home, this person...

Me at home...

During work/study today, this person...

At work, I...

The people next to him/her...

This patient's goal...

The treatment of migraine...

With him/her, I feel...

From my relationship with the patient, I learned...

For tomorrow I would like that I...

For tomorrow I hope he/she...

Thank you for your time, energy and attention. We ask you one last question: How did you feel about writing your experience?
