# Supplementary material for: Narrative Medicine to integrate patients’, caregivers’ and clinicians’ migraine experiences: the DRONE multicentre project
Source: Neurol Sci. 2021 Apr 15;42(12):5277–88. doi: 10.1007/s10072-021-05227-w (PMC8047556; doi:10.1007/s10072-021-05227-w)
Supplement: Supplementary file 3 — (PDF 99 kb) [file 10072_2021_5227_MOESM3_ESM.pdf]

Narrative Medicine to integrate patients', caregivers' and clinicians' migraine experiences: the DRONE multicentre project.

Journal: *Neurological Sciences*

Maria Clara Tonini, Alessandra Fiorencis*, Rosario Iannacchero, Mauro Zampolini, Antonietta Cappuccio, Raffaella Raddino, Elisabetta Grillo, Maria Albanese, Gianni Allais, Marco André Bassano, Filippo Brighina, Terenzio Carboni, Fabio Frediani, Licia Grazzi, Carmela Mastrandrea, Franca Moschiano, Maria Gabriella Poeta, Angelo Ranieri, Renato Turrini, Maria Giulia Marini.

*Corresponding author: Alessandra Fiorencis, Fondazione ISTUD – via Paolo Lomazzo 19, 20124 Milano, Italy. Tel. +39 0323 933 801, Mobile +39 3420499824, e-mail: afiorencis@istud.it. ORCID ID https://orcid.org/0000-0001-9859-5070

Supplement 3

3.1. Narrative from a migraneur

Before the first migraine attack, I was a quiet, studious, sensitive, responsible, responsible, loyal to duty... A "model" child. Then I suddenly started having the first attacks. I felt weak. The first attacks were violent and sudden.

I grew up fast... I was always the tallest... So much so that, at first, they thought that migraine was linked to growing up.

During the attacks, I felt fragile. My parents used to pamper me and tried to "justify" each attack: "Too cold... not enough sleep... bad digestion". They wanted to give answers to the sudden change in my life... Moving from physical "well-being" to a precarious condition was not easy for a child. With other people, I continued to be myself, vulnerable but always eager for warmth and relationships. In my activities, I had to deal with a new condition that risked compromising my normality, my everyday life and my childhood dreams. I often felt sick at school, and my parents had to come and pick me up, I couldn't attend a party. The onset of the migraine ended my dance lessons... We thought that the attacks were due to sinusitis and that sweating and fatigue would not do me any good. I wanted to study and be like the other children/teens... I wouldn't have let this new uncomfortable travelling companion get in my way. Living had become "complicated"... I understood that making plans, even the most ordinary ones, was often impossible.

I decided to go for a visit because I was fed up with being "under attack"! Many specialists were consulted: ENT specialists, ophthalmologists, neurologists... Lots of treatments, no benefits. Often, they proved to be approximate, not very sensitive and not very inclined to listen. They told me that I would have to live with migraine or wait for pregnancy, menopause to get better... The "worst" said to me that I was somatising. The treatments were traditional and experimental, but all ineffective.

Today I feel "stronger" in my fragility and resilient. Migraine is always lurking, unfortunately. It is a part of me... Perhaps, it is the part of me who rebels when she can't take it anymore.

Before a migraine attack, I am waiting for it to come... And I am terrified. I try, however, to

fully enjoy the periods of pause between one episode and another. During the attack, I do not only deal with the pain, but also with myself. Today I can say that migraine has certainly conditioned and limited my life, but it has allowed me to get to know myself better, to listen to myself, to understand myself and to love me more.
Afterwards, it's time to lick your wounds, get up and go back to "living". My body has felt and suffers from these years of pain... Bad posture, gastrointestinal problems, dark circles under my eyes!
I know that it is not easy to be around me. My daughters have suffered and suffer from my condition. The others, some understand me and are sincerely close to me; some pity me, and some do not believe me. I have learned to make differences, to choose. I know that I have to spend my time well and share it only with authentic people. In my activities, for someone like me who grew up with a strong sense of duty, this life suspended between one attack and another is a real undertaking, made of renunciations and departures!
I simply want to feel better! Living is a daily challenge. Treatment is perhaps more effective, and doctors are finally competent and empathic. I wish to do not have migraines anymore! And I don't want to hear "It'll pass... It's just a headache!".
Migraine has given me an awareness of my strength and my potential.

3.2. Narrative from a caregiver

There has never been a "life before migraine". When I met my wife, she was 18 years old and already suffering from migraine. This affliction never left her. I felt and still feel powerless and unable to give her relief. She felt and feels strongly uncomfortable, with little desire to relate to other people. The first attacks were almost always nocturnal, upon waking up and even during work. In any case, they were disabling, but she rarely denied her daily obligations (home, work, children...). She used to tell me that despite the discomfort she couldn't refuse to go to work and only in some instances she used to go to bed in the dark, waiting for the "dark evil" to pass.
Needless to say, how much social life we had to give up, despite the desire and pleasure of doing so. In activities that required an obligation to be present (e.g. growing children, work) she was never spared. I wanted to replace her... Living was and still is today an eternal compromise between what we would like to live fully and many renunciations. She has always undergone specialist visits (even outside our region), but with very few results. The doctors were renowned professors, researchers, experts in the field, without neglecting attempts with non-traditional medicine. They said we had to believe in the goodness of medications, but with no results and as a last resort (someone) even came up with anxiolytics. The treatments were divided between drugs that had to be taken during the attack and others of prevention/maintenance.
I can say without any denial that there are no resolutory strategies in the case of my wife. Acupuncture has also been tried in two or three attempts but abandoned because of the unbearable reflex pain. Today she feels without any hope for a solution and at the same time the conviction that this pathology can only be reacted to with an attitude of "peaceful coexistence".
Migraine is a pathology that cannot be cured, but at the same time offers excellent profit margins because of its indeterminateness, especially to the great luminaries/specialists who unfortunately speculate mercilessly on the lives and "wallets" of these unfortunate people.
The migraine attack is almost always preceded by flickery auras. She says: "I'm getting an attack". During the attack, she sometimes delays in counteracting it pharmacologically, in

the belief that it has now become pharmacoresistant. Afterwards, there are long hours, occasionally half days or whole days, before she feels... "clean".
I would like this pathology to be recognised as disabling and normalised as such in all regions of Italy and beyond. To live is to know how to give up, and to live with pain. I would not like it to be further speculated on as it has been done so far with palliative drugs and expensive specialist centres.

Migraine has sometimes taken away the pleasure of being well as a couple and with other people. Migraine has given us the awareness that it is a pathology that is not fully recognised as not demonstrable, which places the individual suffering in a condition of perennial contradiction.
After having lived an entire life next to a person suffering from migraine, I can only say that it will remain a real challenge for the future to be able to fight it and why not... win it.

3.3. Parallel chart

She always showed up at the clinic alone, despite her age. I was astonished that none of her family (two daughters) could accompany her; but they were always at work, and she didn't want to disturb them with her headache, which had been accompanying her for almost a lifetime, like a friend.
The patient told that she had heard about the headache clinic and had insisted with her doctor that she should be examined. She was a kind lady with a sharp, penetrating, strong-willed look. But she aroused great tenderness and the desire to understand how to help her.
She had suffered from intense, long-lasting and frequent migraine attacks until the menopause, then they had thinned out and for about ten years sporadically showed up. They were less severe, shorter in duration, but when they did occur, they were always annoying. She asked me if I had any new medicine to make them disappear completely. To get rid of this boring friend.
She had never worked; she had always taken care of her family. During the migraine attacks, even if intense, she tried hard not to leave out her duties of looking after her daughters, grandchildren and the severe illness of her husband, who had been missing for two years. She had never had prophylaxis therapy and blocked the attacks with paracetamol and recently with ibuprofen. She went to bed when she could, in the dark for a few hours, also because she felt exhausted. But she also felt tired of the emptiness left by her husband.
I felt a great tenderness; not only because of the way she told without emphasis of her physical pain – given by headache and a series of disorders due to osteoporosis and widespread arthrosis – but also a psychological distress. Behind her discreet story, there was a lot of suffering, a desire to be understood, to talk, to pay attention, to fill a void. Perhaps I was saying to myself that she had given a lot of attention to her family, taking it away from herself; she had fought for the well-being of others and not for herself. Now here, she was asking for listening.
I collected her medical history; I did the neurological examination; I prescribed an MRI Encephalus (which she had never done) and which I considered necessary, considering that at that age headache was still present. I prescribed her a therapy with Magnesium and a supplement and requested a check-up.
The MRI of her brain was compatible with a long history of migraine and her hypertension. When she came back, she asked me if she could come more often to talk, to "chat" not only about her headache, which after all was not so ungovernable. But certainly, this was a way out of her unhappiness. The next time she came to talk about osteoporosis, then because she was not resting, then because of her pains. Every time she hugged me and told me that I was an angel. She brought me cakes or

homemade pasta. She didn't talk to me anymore about his headache, but about anything else. Every time I saw her name on the waiting list, I told myself hopefully everything would be all right; and everything was fine, she came to greet me.
She wanted some attention, some affection that we doctors are not always willing to give: we are afraid of it, we fear to lose our role. And letting myself go to a non-traditional, more affective, more open to that hug made me feel good, it gave an added value to what I was doing and the energy to face the next visits. I told myself to go on like this, don't be afraid. The angel is not me, but she does, my "tender" patient.
