# Supplementary material for: Narrative Medicine to integrate patients’, caregivers’ and clinicians’ migraine experiences: the DRONE multicentre project
Source: Neurol Sci. 2021 Apr 15;42(12):5277–88. doi: 10.1007/s10072-021-05227-w (PMC8047556; doi:10.1007/s10072-021-05227-w)
Supplement: Supplementary file 4 — (PDF 126 kb) [file 10072_2021_5227_MOESM4_ESM.pdf]

Narrative Medicine to integrate patients', caregivers' and clinicians' migraine experiences: the DRONE multicentre project.

Journal: *Neurological Sciences*

Maria Clara Tonini, Alessandra Fiorencis*, Rosario Iannacchero, Mauro Zampolini, Antonietta Cappuccio, Raffaella Raddino, Elisabetta Grillo, Maria Albanese, Gianni Allais, Marco André Bassano, Filippo Brighina, Terenzio Carboni, Fabio Frediani, Licia Grazzi, Carmela Mastrandrea, Franca Moschiano, Maria Gabriella Poeta, Angelo Ranieri, Renato Turrini, Maria Giulia Marini.

*Corresponding author: Alessandra Fiorencis, Fondazione ISTUD – via Paolo Lomazzo 19, 20124 Milano, Italy. Tel. +39 0323 933 801, Mobile +39 3420499824, e-mail: afiorencis@istud.it. ORCID ID https://orcid.org/0000-0001-9859-5070

Supplement 4

Metaphors used by participants to describe migraine: distribution and examples.

**Limitation or still life**	**20% patient narratives**	—*The remains of a sea storm on the shoreline: broken shells, dead and putrid fish, worn wood, plastic bags, broken and worn shoes...* (Patient 001)<br>—*A butterfly in a cage*. (Patient 028)<br>—*Fog*. (Patient 100)
	**38% caregiver narratives**	—*Her migraine is a lightless tunnel.* (Caregiver 024)<br>—*A cloudy sea*. (Caregiver 002)<br>—*A withered rose*. (Caregiver 018)
	**25% parallel charts**	—*Her migraine is a cage*. (Parallel chart 031)<br>—*An impassable wall*. (Parallel chart 003)<br>—*A black hood*. (Parallel chart 002)
**Malignant nature or entity**	**31% patient narratives**	—*I would associate migraine with a tsunami. At first, you feel that it is coming. When it arrives, it completely overwhelms you [...]; when it goes away, it leaves "aftermaths", the result of its passage. [...] Despite all efforts, it still comes and overwhelms you*. (Patient 066)<br>—*The infernal hunger of Count Ugolino intent on biting his damned comrade's head off*. (Patient 010)<br>—*The earth rising as if breathing frantically*. (Patient 103)
	**25% caregiver narratives**	—*Her migraine is a room saturating with gas and ready to explode*. (Caregiver 014)<br>—*A dark cloud*. (Caregiver 023)<br>—*Lightning in clear skies*. (Caregiver 017)
	**30% parallel charts**	—*His migraine is a periodic storm*. (Parallel chart 006)<br>—*An animal that paralyses and frightens her*. (Parallel chart 033)<br>—*A series of flashes and sparks that stuns and frightens her*. (Parallel chart 024)
**Hammer, pressure, or pulsing**	**30% patient narratives**	—*Migraine is like a clamp tightening my head*. (Patient 035)

		—*I am lying on the bed. My body is gathered in fetal position, my head held in my hands or surrounded by my arms. A hammer beats against my eye as if it would to fracture my skull, another one beats incessantly on top, in the middle of my head. Sometimes simultaneously. One is enough to make you think of nothing else*. (Patient 084)<br>—*My migraine is a vice that compresses my head and generates me within the pulsating blows*. (Patient 063)
	**13% caregiver narratives**	—*Her migraine is a hammer*. (Caregiver 012)<br>—*A huge weight on his head*. (Caregiver 006)<br>—*She was feeling as if her head were in a vice*. (Caregiver 011)
	**34% parallel charts**	—*His migraine is a beating heart in the head*. (Parallel chart 016)<br>—*A pincer*. (Parallel chart 034)<br>—*A balloon that swells painfully in the skull and produces a continuously worsening pain*. (Parallel chart 027)
**Stabbing and pain**	**18% patient narratives**	—*Migraine is like a knife stuck in the recess above the eyelid*. (Patient 044)<br>—*I would say that Munch's "The Scream" well represents my feelings*. (Patient 040)<br>—*A form of torture with which it is impossible to live with*. (Patient 045)
	**25% caregiver narratives**	—*Her migraine is an endless screw*. (Caregiver 019)<br>—*Way of the Cross*. (Caregiver 022)<br>—*Desperation*. (Caregiver 010)
	**7% parallel charts**	—*His migraine is a screwdriver*. (Parallel chart 018)<br>—*A blade that penetrates the brain*. (Parallel chart 044)<br>—*It's like having a nail in your head*. (Parallel chart 040)
